# An unusual case of complete pathological response to Paget’s disease of the breast

**DOI:** 10.1093/jscr/rjac106

**Published:** 2022-04-11

**Authors:** Daisy Okonofua, Chien Lin Soh, Habib Tafazal

**Affiliations:** North West Anglia NHS Foundation Trust, Breast Unit, Peterborough City Hospital, Peterborough UK; School of Clinical Medicine, University of Cambridge, Cambridge, UK; North West Anglia NHS Foundation Trust, Breast Unit, Peterborough City Hospital, Peterborough UK

**Keywords:** breast cancer, oncology, radiology, imaging

## Abstract

Mammary Paget’s disease is a rare form of breast cancer present in ~0.5–2.8% of breast cancers. The patients have a poorer prognosis, and usually, the appropriate therapy is based on the pathologic findings of the mass and axillary staging. This report adds the outcomes of Paget’s disease following neoadjuvant chemotherapy to the literature by description of a case of a 48-year-old patient with Paget’s disease who had a complete pathological response to Paget’s disease of the breast following neoadjuvant chemotherapy.

## INTRODUCTION

Mammary Paget’s disease is a rare form of breast cancer which was first described by Velpeau in 1856 and was further characterized by Sir James Paget in 1874 as nipple ulceration with an associated carcinoma. It is reported to be present in ~0.5–2.8% of breast cancers.

The disease is characterized by an eczematous lesion and ulceration that affects the nipple and areola. An underlying intraductal spread or invasive carcinoma is commonly detected by histopathological examination. The underlying mass is often an invasive cancer with a high risk of nodal metastases. The patients have a poorer prognosis, and usually, the appropriate therapy is based on the pathologic findings of the mass and axillary staging. Mastectomy with or without axillary lymph node dissection has long been regarded as the standard therapy for Paget’s disease even in the absence of other clinical signs of invasive malignancy. Even with standard surgical treatment, the risk of recurrence is reported to be high. We report a case of complete pathological response to Paget’s disease of the breast following neoadjuvant chemotherapy.

## CASE PRESENTATION

A 48-year-old lady presented to the breast clinic with complaints of pain in her right breast and associated right nipple bleeding for 3 weeks. She had no family history of breast cancer. She was a non-smoker and did not take any regular medications. Her co-morbidities included Hepatitis C.

**Figure 1:**
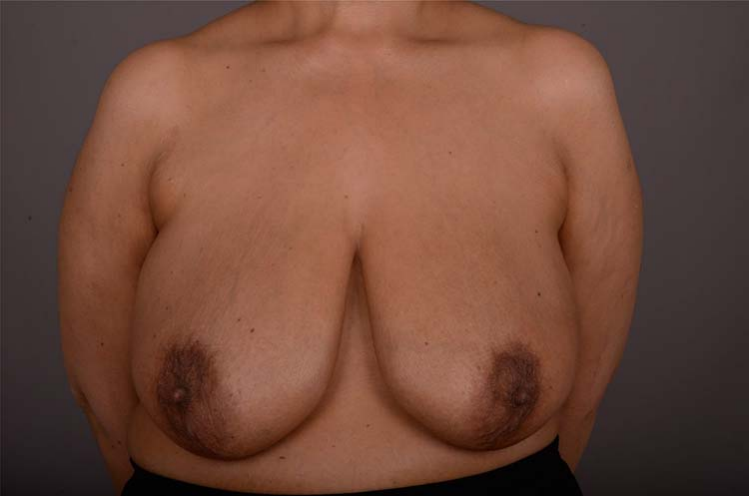
View of both breasts at initial presentation.

**Figure 2:**
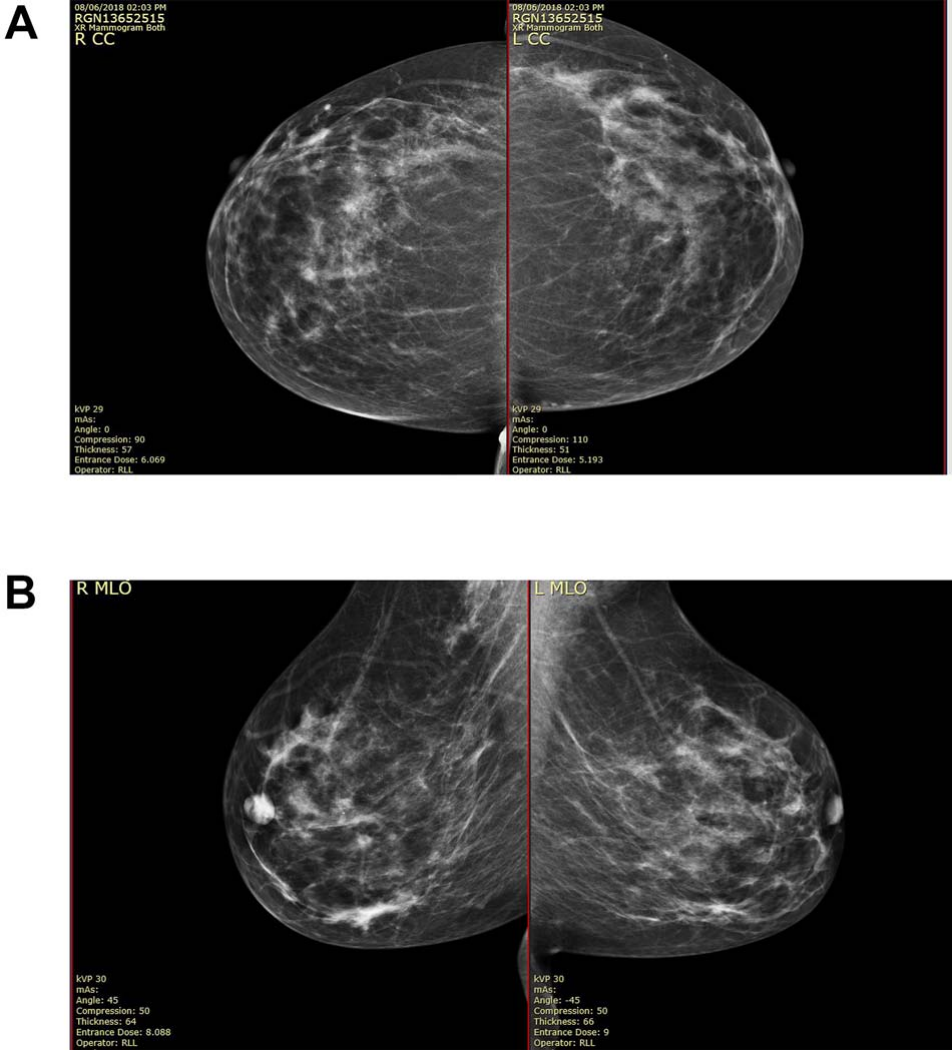
(**A**) Mammography showed widespread segmental distribution of pleomorphic calcification on the right breast measuring up to 11 cm; (**B**) mammography showed widespread segmental distribution of pleomorphic calcification on the right breast measuring up to 11 cm.

**Figure 3:**
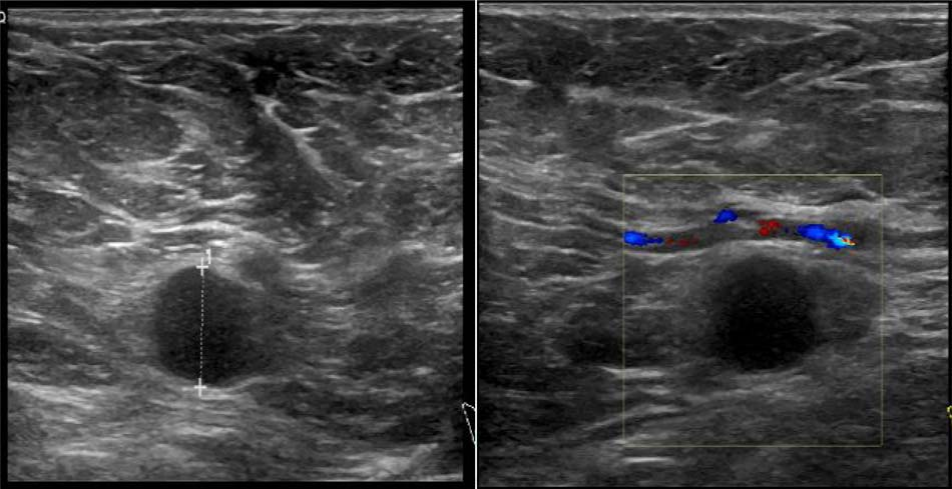
US findings of abnormal lymph node in right axilla.

On clinical examination, there were tender nodularities in the upper outer quadrant of the right breast. The nipple looked thick and tender at the nipple tip, with punctate bleeding from different sites (P3) (Fig. 1). Examination on the left breast and both of the axillae were unremarkable. The mammogram reported segmental distribution of pleomorphic calcification on the right breast, which was widespread and was measuring up to 11 cm from the nipple posteriorly. The appearances of the calcifications were suspicious for malignancy (M4). There were benign appearing calcifications on the left breast as well (M2) (Fig. 2). On ultrasound (US), there was no discrete mass or suspicious feature seen in the right upper outer quadrant. However, there was a 10-mm deep-seated, round, abnormal-looking lymph node on the right axilla (U4), which could not be biopsied at the time due to a pulsating blood vessel overlying it [1] (Fig. 3).

**Figure 4:**
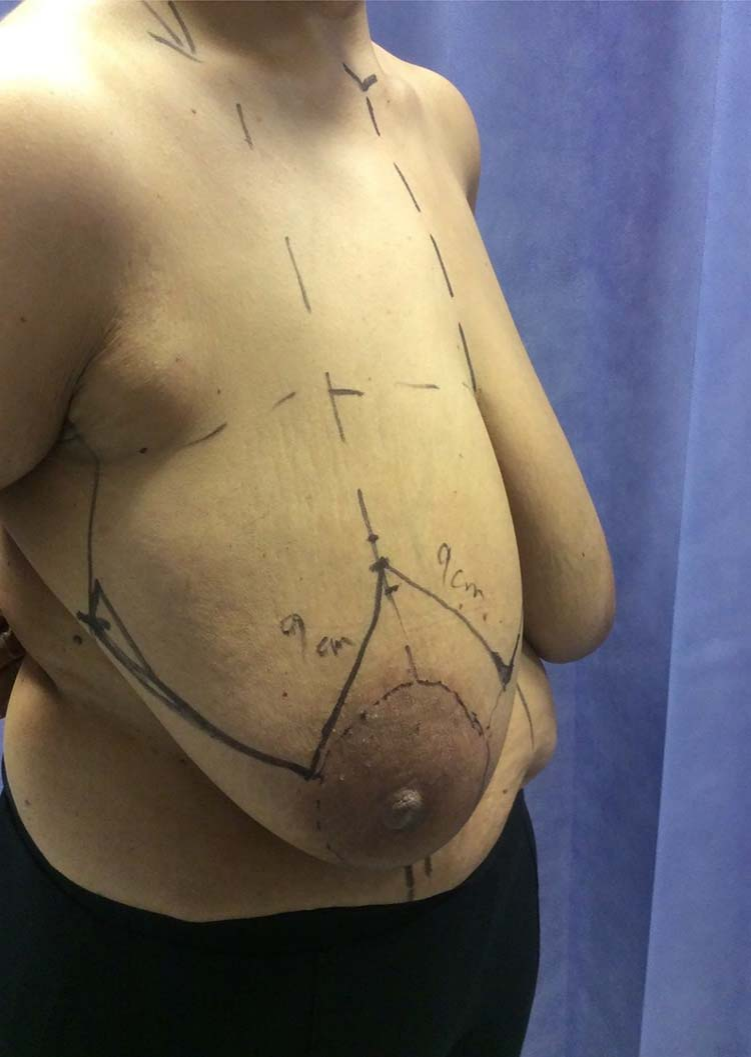
Pre-op view.

**Figure 5:**
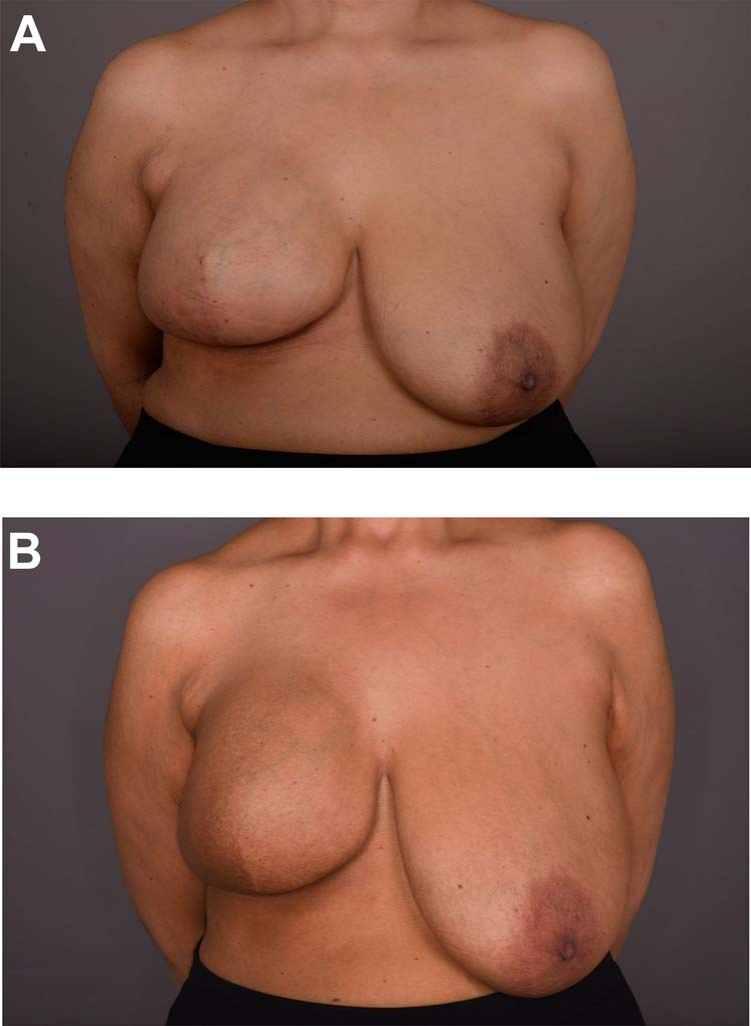
(**A**) 3 months post-op; (**B**) post-radiotherapy.

She subsequently had a stereotactic core biopsy of the mammographically abnormal region in the right breast and an USS-guided core biopsy of the right axillary mass. A punch biopsy was taken from the nipple. Biopsy results showed Paget’s disease of the right nipple with Grade 2 invasive ductal carcinoma of the right breast, ER-positive, PR-positive and HER2-positive. There was associated high-grade DCIS. Results from the right axilla were consistent with metastatic breast adenocarcinoma.

The decision of the multidisciplinary committee (MDT) was for neoadjuvant chemotherapy, staging computed tomography and bone scans, followed by nipple sacrificing mastectomy, axillary node clearance and immediate reconstruction. The staging scans showed no evidence of distant metastatic disease. She was subsequently commenced on a chemotherapeutic regimen of Taxotere, Pertuzumab and Transtuzumab, followed by Epirubicin and Cyclophosphamide, for a total of 21 weeks by the medical oncology team.

Following completion of neoadjuvant chemotherapy, she had a right nipple sacrificing mastectomy and right axillary nodal clearance with prepectoral implant reconstruction (Figs 4 and 5). Final histology showed complete pathological response with no residual malignancy. All 17 lymph nodes showed no fibrosis nor metastatic deposits. The sections from the nipple showed non-specific chronic inflammatory changes, likely indicating regression of Paget’s disease secondary to neoadjuvant chemotherapy. The MDT further recommended radiotherapy to chest wall and continuation of adjuvant Herceptin.

## DISCUSSION

Paget’s disease of the breast is a rare nipple pathology which often presents alongside a breast carcinoma. The patient may present with early manifestations of scaling and redness on the nipple, which is often misdiagnosed as eczema or inflammatory reactions [2]. Later advancement of Paget’s disease may result in a round plaque with rashes and ulceration with or without discharge. A unilateral lesion is more common than bilateral cases, although some cases of bilateral Paget’s disease have been described in the literature [3]. The majority of Paget’s disease is associated with an underlying DCIS or invasive carcinoma [4], although it may also present without underlying malignancy [5]. Many theories have been proposed regarding the pathogenesis of Paget’s disease, which remains controversial in the literature.

When Paget’s disease is suspected, further radiological and histopathological assessment is required to assess whether there is underlying breast carcinoma. Imaging can also aid in management of the disease, whether surgery or adjuvant therapy is indicated. Breast ultrasonography, mammography and magnetic resonance imaging can be used in combination to detect any underlying malignancy. Histopathological assessment is used to determine the presence of intraepidermal Paget’s cells, glandular epithelial cells with clear cytoplasm and enlarged nuclei characteristic for the disease. Hormonal receptors, HER2 and Ki-67 expression may differ among each case; which can target therapy for each patient [6].

Surgery is the mainstay of treatment of Paget’s disease of the breast. Mastectomy with or without axillary dissection has been traditionally offered to patients; however, breast-conserving therapy has comparable clinical outcomes, such as equivalent disease-free and overall survival rates, with patients who have DCIS or without an underlying tumor [7]. Sentinel lymph node biopsy is offered to patients in clinically node-negative cancer, however, it has not shown any clinical outcome benefit between patients who did not undergo SLN in a database review study [8].

Adjuvant therapy is often then decided on a case-by-case basis. Adjuvant endocrine, radiation and chemotherapy is offered to all patient cohorts [9]. Neoadjuvant chemotherapy is a recent development which is becoming more popular against breast cancer and has been shown to achieve better clinical outcomes [10]. Pathological complete response is defined as the elimination of all invasive cancer following completion of neoadjuvant chemotherapy. This has been shown to increase the overall survival. Pathological complete response of mammary Paget’s disease, which we report in our patient, has not been previously reported in the reviewed literature. However, there has been a reported positive response to extramammary Paget disease in a patient who received docetaxel as neoadjuvant chemotherapy [11].

## CONCLUSION

This report adds the outcomes of Paget’s disease following neoadjuvant chemotherapy to the literature. Further studies are needed to further evaluate the predictors and to identify the group of patients with Paget’s disease who would most likely benefit from receiving neoadjuvant chemotherapy.

## PATIENT CONSENT STATEMENT

The patient had provided informed, written consent for the publication of the case report and photography.

## CONFLICT OF INTEREST STATEMENT

The authors declare no competing financial or non-financial interests.
